# Effect of an Antibacterial Monomer on the Antibacterial Activity of a Pit-and-Fissure Sealant

**DOI:** 10.1371/journal.pone.0162281

**Published:** 2016-09-29

**Authors:** Fan Yu, Haohan Yu, Pingting Lin, Yan Dong, Ling Zhang, Xiang Sun, Zhengya Liu, Huihui Guo, Li Huang, Jihua Chen

**Affiliations:** 1 State Key Laboratory of Military Stomatology & National Clinical Research Centre for Oral Disease & Shaanxi Key Laboratory of Stomatology, Department of Prosthodontics, School of Stomatology, Fourth Military Medical University, Xi’an, China; 2 State Key Laboratory of Military Stomatology & National Clinical Research Centre for Oral Disease & Shaanxi Engineering Research Center for Dental Materials and Advanced Manufacture, Department of VIP Dental Care, School of Stomatology, Fourth Military Medical University, Xi’an, China; 3 State Key Laboratory of Military Stomatology & National Clinical Research Center for Oral Disease & Shaanxi Key Laboratory of Stomatology, Department of Operative Dentistry & Endodontics, School of Stomatology, Fourth Military Medical University, Xi’an, Shaanxi, China; 4 State Key Laboratory of Military Stomatology & National Clinical Research Center for Oral Disease & Shaanxi International Joint Research Center for Oral Diseases, Department of General Dentistry and Emergency, School of Stomatology, Fourth Military Medical University, Xi’an, Shaanxi, China; Institute of Materials Research and Engineering, SINGAPORE

## Abstract

Resin-based pit-and-fissure sealants are often used to form a barrier on the occlusal surface of molars to treat caries lesions; however, bacteria can remain in the pit and fissures without detection, increasing the risk of secondary caries. Sealants with antimicrobial properties or microbial repellent actions might be advantageous. The aim of this study was to assess the inhibitory effect of a 2-methacryloxylethyl dodecyl methyl ammonium bromide (MAE-DB)-incorporated sealant against *Streptococcus mutans*. MAE-DB (4% wt) was incorporated into a commercially available sealant, Eco-S resin-based pit-and-fissure sealant (Vericom Co., Ltd., Korea); a sealant without MAE-DB served as a negative control, and Clinpro™ Sealant (3M™ ESPE™), a fluoride-releasing resin, was used as a commercial control. The effects of the cured sealants and their eluents on the growth of *S*. *mutans* were determined according to colony-forming unit counts and metabolic tests. The effects of the cured sealants on the adherence and membrane integrity of *S*. *mutans* were investigated using confocal laser-scanning microscopy (CLSM) in conjunction with fluorescent indicators. Compared with the negative control and commercial control, the cured MAE-DB-incorporated pit-and-fissure sealant exhibited a significant inhibitory effect on the growth of *S*. *mutans* (P < 0.05), whereas the eluents did not show any detectable antibacterial activity. The commercial control also showed no detectable bactericidal activity. Moreover, the aged experimental material retained its property of contact inhibition of biofilm formation. The fluorescence analysis of CLSM images demonstrated that the cured MAE-DB-incorporated sealant could hamper the adherence of *S*. *mutans* and exert a detrimental effect on bacterial membrane integrity. The incorporation of MAE-DB can render a pit-and-fissure sealant with contact antibacterial activity after polymerization via influencing the growth, adherence, and membrane integrity of *S*. *mutans*. Therefore, MAE-DB-containing pit-and-fissure sealant shows promise for preventing or controlling dental caries on occlusal pit and fissures of molars.

## Introduction

Caries lesions develop from biofilm accumulation and sugar exposure on the dental surface, and is one of the most prevalent dental diseases worldwide [1]. The occlusal pit and fissures, with invaginations or irregularities, are generally too narrow (about 0.1 mm wide) for a standard toothbrush and saliva to reach for effective cleaning, easily allowing bacteria and food to be mechanically retained; thus, 85% of all dental caries occur on the occlusal surface, especially in young, permanent teeth [2, 3]. Accordingly, the sealing of pits and fissures with bondable materials is a widely accepted strategy for the prevention of occlusal caries of the molars and premolars, as well as for incipient carious lesions treatment [4, 5]. A mechanical barrier is created between the oral environment and the occlusal surfaces of the teeth, thus preventing bacteria and food penetration into the vulnerable recesses of the occlusal surfaces [2, 6]. Indeed, there are ample evidences from reviews suggest that sealants are effective to prevent or arrest the progression of noncavitated carious lesions compared with the nonuse of sealants[7–9].

Glass-ionomer cements and bondable resin-based pit-and-fissure sealants are the main materials recommended for this purpose. However, there is no definitive conclusion as to whether glass-ionomer cements are equally effective as resin-based dental sealants in preventing occlusal dental caries in permanent or primary teeth [10]. The main shortcoming of resin-based materials is the potential for microleakage around the sealants because of polymerization shrinkage, occlusal forces, and aging, thus causing damage to the physical barrier, leading incomplete isolation [11, 12]. The invasion of bacteria and oral fluids via the microleakage may decrease the efficacy of occlusal caries prevention[13]. Studies have also found that resin-based materials can accumulate more biofilms than other materials, thereby increasing the risk of carious progress [14–16]. In addition, during the treatment of incipient carious lesions, under some circumstances, the operator may be uncertain as to whether the remaining debris, pellicles, or bacteria present in the pits and fissures are completely removed [17, 18]. Thus, use of a material with antibacterial effects would be beneficial in this procedure when caries is inadvertently left in the surface to be sealed and would strengthen the defense mechanism of the later against the bacteria[19–21].

There have been various attempts to develop antibacterial dental materials. Many researchers have modified resin-based pit-and-fissure sealants by the addition of soluble antimicrobials such as fluoride and chlorhexidine (CHX) [22, 23]. The addition of fluoride to pit-and-fissure sealants has been applied widely owing to its beneficial effects in reducing demineralization, enhancing remineralization, and inhibiting microbial metabolism and plaque formation [24]. However, it is impossible to achieve strict control of the release kinetics, and fluoride release will decrease with time [25] and may be not sufficient for a maximal antibacterial effect[26]. Therefore, it is currently not clear whether a fluoride-containing sealant has effective antibacterial activity, and available evidence does not provide a definitive conclusion as to whether such a sealant is actually superior for preventing caries compared to a sealant without fluoride [27]. In addition, resins that do release fluoride ions are relatively weak and cannot be used in large stress-bearing restorations [28]. CHX, with its broad-spectrum antibacterial activity and low cytotoxicity, has also been widely used for caries prevention [29, 30], and CHX-containing dental composites have been developed and investigated. Although CHX is similar to fluoride, the mechanical or physical properties of CHX-containing resins may be compromised by the constant release of antibacterial agents, especially due to the porous surface that is formed, which may lead to poor wear resistance and could increase the potential for staining and bacterial biofilm accumulation [31]. Indeed, one study showed that application of CHX to pit-and-fissure sealants increased microleakage in sealed teeth [23].

In recent year, polymeric derivatives have been applied to endow a medical material with antibacterial activity, such as Polyethylenimines (PEIs)[32], Quaternary ammonium[33], Poly(L-lactide)-based[34], Porphyrin-based[35], Polyphenols- and Catecholamines-based polymers[36]. Quaternary ammonium monomers(QAMs), such as 12-methacryloyloxydodecylpyridinium bromide (MDPB) [37–39], methacryloxylethyl cetyl dimethyl ammonium chloride (DMAE-CB) [40, 41] and 2-methacryloxylethyl dodecyl methyl ammonium bromide (MAE-DB) [42, 43], have been introduced to endow a dental material with antibacterial activity. The QAMs can be chemically immobilized within a resin matrix and the antibacterial mechanism of QAMs is due to their capability of causing bacteria lysis by binding to bacterial membranes [44]. Our previous study demonstrated that MAE-DB-modified resin materials exerted excellent long-lasting antibacterial activity and an antibiofilm effect against *Streptococcus mutans* [43]. However, its potential effect on pit-and-fissure sealants is not clear so far.

In this study, an experimental pit-and-fissure sealant was fabricated by incorporating MAE-DB into a commercial resin-based sealant material, and its antibacterial activity against *S*. *mutans* and were evaluated. Our hypothesis was that the mobilized MAE-DB in the resin-based sealant would exhibit effective and long-lasting antibacterial activity against *S*. *mutans*.

## Material and Methods

### Sample preparation

**Fig 1:**
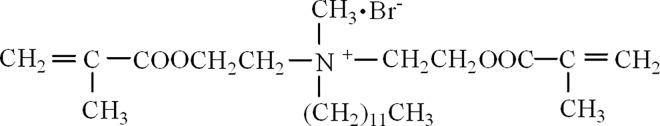
Structure of MAE-DB.

The structure of MAE-DB is presented in Fig 1. A commercially available resin-based pit-and-fissure sealant, Eco-S (Vericom Co., Ltd., Korea), was used as the parent material for antibacterial functionalization. As the test material, we used Eco-S supplemented with 4 wt% of the cationic monomer MAE-DB. Eco-S without the MAE-DB monomer served as the negative control. We used Clinpro™ Sealant (3M™ ESPE™, hereafter referred to as Clinpro), a fluoride-releasing resin, as the commercial control.

Twenty-four-well plates (Costar, Corning, Lowell, MA, USA) were used as a mold for specimen preparation. We spread the sealant from each group onto the round dents on the bottom of the 24-well plates, which were irradiated with curing light (Dentsply QHL 75, Milford, DE, USA) for 20 s to obtain specimens with a diameter of 8 mm. All specimens were sterilized with ethylene oxide gas, followed by degassing for more than 48 h.

To simulate the aging process, five specimens of each group were placed in wells of a 24-well plate containing 1 mL of distilled water, which was replenished every 3 days. After aging for 6 months at 37°C, the specimens were retrieved, sterilized, and subjected to the following experiments

### Bacterial strains and culture conditions

*Streptococcus mutans* strain UA159 provided by The Endodontics Department and Microbiology Department of the Fourth Military Medical University was cultured overnight at 37°C in brain heart infusion (BHI) broth (Difco, Detroit, MI, USA) in an anaerobic atmosphere (90% N_2_, 5% CO_2_, and 5% H_2_). We adjusted the bacterial suspension to 1 × 10^5^ colony-forming units (CFU)/mL for further use.

### Inhibitory effect of cured materials on the growth of *S*. *mutans* before and after the aging process

The prepared disks for testing with or without aging were placed in a 24-well plate with 2 mL of BHI broth. Then, 20 μL of the diluted *S*. *mutans* suspension was added to each well. After 24 h of anaerobic culture, the bacterial cells were collected by vortexing the specimen vigorously in 9.99 mL of BHI at maximum speed for 2 min using a vortex mixer (Fisher Scientific, Pittsburgh, PA, USA). The bacterial suspension of each specimen was serially diluted, inoculated on a BHI agar plate, and incubated for 1 day at 5% CO_2_ and 37°C to determine the total number of CFU recovered. Five specimens of each group were subjected to CFU counts.

For the metabolic test, Cell Counting Kit-8 (7Sea Biotech, Shanghai, China) was used according to the manufacturer instruction. In brief, the bacterial suspensions obtained from the disks were prepared as mentioned above. Two-hundred microliters of the bacterial suspensions obtained from the disks were transferred to a 96-well plate, and 20 μL of CCK-8 dye solution was added to each well and incubated at 37°C in 5% CO_2_ for 2 h. The absorbance at 450 nm was measured using a microplate reader (Spectra Max M5, Molecular Devices, Sunnyvale, CA, USA). Each sample was assayed in triplicate, and an average value was calculated for each sample.

### Influence of material eluents on the growth of *S*. *mutans*

The prepared disks of each group, with or without aging, were spread onto the bottom of the wells in a 24-well plate (Costar, Corning Inc., Corning, NY, USA) and irradiated for 20 s in an anaerobic chamber. Subsequently, each well was rinsed with 1 mL of sterile distilled water, 250 μL of BHI, and 20 μL of distilled water to prepare the sealant eluent. After incubation for 24 h at 37°C, the eluents were transferred to a 24-well plate to evaluate their influence on bacterial growth according to CFU counts and the results of the metabolic test, as described above.

### Confocal laser-scanning microscopy (CLSM) analysis of bacterial growth

*S*. *mutans* coated on sample disks with or without aging were analyzed by CLSM. After 24-h incubation, the disks coated with biofilms were washed three times with sterile saline to remove loose bacteria, and the remaining bacteria were stained using the Live/Dead BacLight Bacterial Viability Kit L7012 (Molecular Probes, Eugene, OR, USA) with 15-min incubation in the dark at room temperature to allow for stain development for image scanning. The samples were rinsed gently with distilled water and observed by CLSM (FluoView FV1000, Olympus, Tokyo, Japan). Excitation with a 488-nm laser revealed the green fluorescence emission of live bacteria, and excitation with a 543-nm laser revealed the red fluorescence emission of bacteria with damaged membranes.

### Statistical analysis

Statistical analyses were performed using SPSS 17.0 software, and judged at a significance threshold level of P < 0.05. One-way and two-way analyses of variance (ANOVAs) were performed to detect the significant effects of the variables (material type and aging status) on CFU count and bacterial metabolic activity. The Tamhane multiple comparison test was used to compare differences between any two groups, with significance assumed at P < 0.05. Standard deviation (SD) values were used as estimates for the uncertainty associated with particular measurements.

## Results

### Inhibitory effect of cured materials on the growth of *S*. *mutans* before and after the aging process

**Fig 2:**
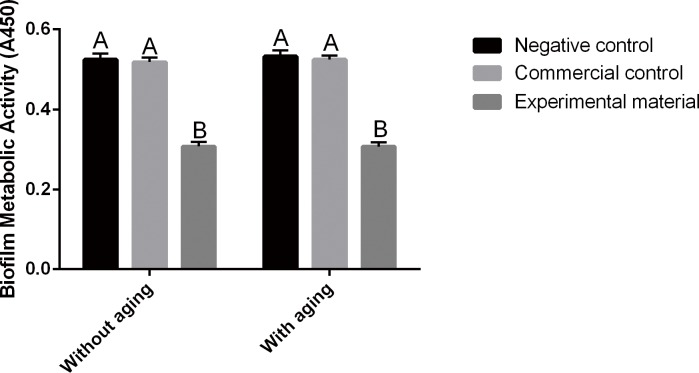
Metabolic activity of *S*. *mutans* on material surfaces (mean ± SD; n = 6) for the negative control, commercial control, and experimental materials, and the corresponding aged samples. Absorbance values were analyzed with one-way and two-way ANOVA at a significance level of 0.05. Values with dissimilar superscript letters are significantly different (P < 0.05). Values with the same superscript letter are not significantly different (P > 0.05).

The antibacterial effects of cured materials on the growth of *S*. *mutans* before and after the aging process are shown in Table 1 and Fig 2. There was a significant difference in bacterial growth on the experimental sealant containing 4 wt% MAE-DB with growth on the negative or commercial control samples (P < 0.001), whereas there was no difference in growth between the negative and commercial control groups (P > 0.05). For each material, the aging process had no significant effect on the CFU counts (P > 0.05). In addition, aged sealants showed the same statistical relationships as their non-aged counterparts.

**Table 1:** Colony-forming units (CFU) counts from S. mutans biofilms on the material surfaces.

Group	CFU	
	Without aging	With aging
Negative control	(6.43 ± 0.75) × 10^8A^	(6.25 ± 0.66) × 10^8A^
Commercial control	(6.09 ± 0.54) × 10^8A^	(5.8 ± 0.66) ×10^8A^
Experimental material	(4.74 ± 0.97) × 10^6B^	(4.83 ± 1.16) × 10^6B^

CFU values represent the mean ± SD of three replicates, and data were analyzed with one-way and two-way ANOVA at a significance level of 0.05. Values with dissimilar superscript letters are significantly different (P < 0.05). Values with the same superscript letter are not significantly different (P > 0.05).

### Influence of material eluents on the growth of *S*. *mutans*

**Fig 3:**
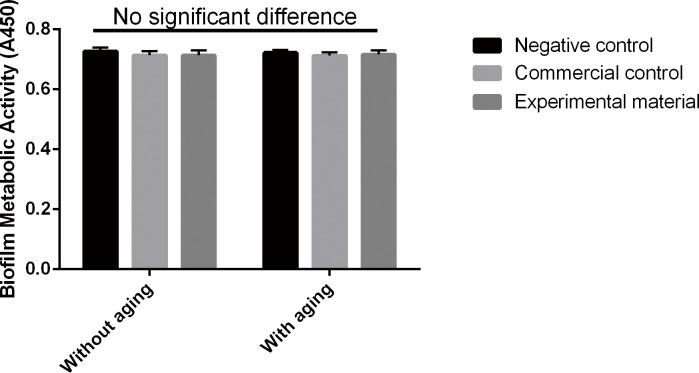
Metabolic activity of *S*. *mutans* cultured with different material eluents. Absorbance values were analyzed with one-way and two-way ANOVA at a significance level of 0.05. No significant differences were observed between any conditions (P > 0.05).

The CFU counts and metabolic activity of *S*. *mutans* cultured with different material eluents are shown in Table 2 and Fig 3, respectively. Two-way ANOVA showed that neither material type nor aging had a significant effect on the CFU count and metabolic activity (all P > 0.05). There were no significant differences among the subgroups regardless of the aging process (all P > 0.05). Moreover, the eluents from the commercial control group and the experimental material did not show a detectable inhibitory effect on *S*. *mutans*.

**Table 2:** Colony forming units (CFU) counts from S. mutans biofilms in the material eluents.

Group	CFU	
	Without aging	With aging
Negative control	(6.79 ± 0.7) × 10^8A^	(6.84 ± 0.53) × 10^8A^
Commercial control	(6.26 ± 0.46) × 10^8A^	(6.55 ± 0.44) ×10^8A^
Experimental material	(6.45 ± 0.61) × 10^8A^	(6.62 ± 0.47) × 10^8A^

CFU values represent the mean ± SD of three replicates, and data were analyzed with one-way and two-way ANOVA at a significance level of 0.05. Values with dissimilar superscript letters are significantly different (P < 0.05). Values with the same superscript letter are not significantly different (P > 0.05).

### CLSM analysis of bacterial growth

**Fig 4:**
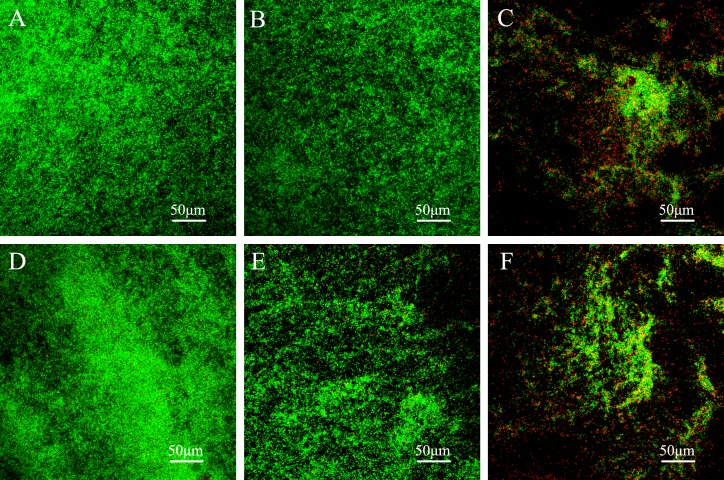
Representative confocal laser-scanning microscope (CLSM) images of live/dead-stained biofilms on the material surfaces. Representative CLSM images of live/dead-stained biofilms after 24 h of anaerobic growth on the tested material surfaces: (A) negative control material, (B) commercial control material, (C) experimental material; biofilms on the corresponding aged samples are shown in D–F. Live bacteria exhibited green fluorescence, and bacteria with compromised membranes exhibited red fluorescence. Scale bars, 50 μm.

Representative live/dead staining CLSM images of the adherent biofilms on resin disks are shown in Fig 4. With the staining kit used in this study, live bacteria produce green fluorescence upon staining with Syto9, and bacteria with compromised membranes produce red fluorescence upon staining with propidium iodide. The specimens of the negative control and commercial control groups with or without aging were fully covered by primarily live bacteria (green), whereas the MAE-DB-incorporated sealant with or without aging showed a lower density of cells and a greater proportion of dead bacteria (red) when compared with the other groups.

## Discussion

The occlusal surfaces of the permanent molars are highly susceptible to caries, accounting for more than two thirds of the total lesions that develop in children; thus, sealing of pits and fissures has proven to be an effective strategy for caries prevention on the occlusal surface [45]. A resin-based sealant is often the first choice material for this purpose, because it is cost-effective, has direct-filling capability and appropriate mechanical properties, and is easy to operate [46–48]. However, residual bacteria may persisted in the prepared cavity, and bacterial penetration via microleakage may increases the risk of subsequent treatment failure[13, 19]; thus, researchers have been dedicated to the study of antimicrobial resin materials. In the present study, the QAM MAE-DB, which has previously shown effective antibacterial activity, was added to a commercial pit-and-fissure sealant and its antibacterial properties were evaluated by a CCK-8 assay, CFU counts, and qualitative analysis with CLSM.

The results of the direct contact test showed that the growth of *S*. *mutans* was significantly inhibited by contact with the sealant containing MAE-DB, compared with the negative control group and commercial control group. After 6 months of aging, the antibacterial activity of the MAE-DB-containing sealant against *S*. *mutans* did not decrease, indicting long-lasting antibacterial capability. The results from this study are consistent with the similar effects reported of a resin-based pulp-capping material containing MAE-DB in our previous study [42]. Since the accumulating effects of long-term bacterial metabolism due to microleakage is the main factor responsible for generating secondary caries, use of a resin-based pit-and-fissure sealant with long-lasting antibacterial activity would be beneficial.

To evaluate whether the observed antibacterial activity was attributable to the bioactive components that are released into the medium, the antibacterial activity of the eluents from the materials was also tested according to CFU counts. In contrast to the direct contact test, no significant difference from the control was detected among the three groups. When a QAM is incorporated into a resin matrix, covalent bonds are formed with the polymer network upon photo-polymerization [31, 49]. Therefore, the antibacterial monomer becomes immobilized in the resin and does not leach out over time, thus providing a durable contact-antibacterial capability [31] and minimizing cytotoxicity [50].

In this study, Clinpro, which contains fluoride as the main antibacterial ingredient, did not show any noticeable antibacterial activity both in the contact test and when tested as elutes, presumably owing to the low concentration of fluoride released from the cured sealant. Clinpro contains an organic fluoride compound, tetrabutylammonium tetrafluoroborate, which could be incorporated into an unpolymerized resin and polymerizes with the resin matrix, therefore rendering it a low-level fluoride release property [51]. Naorungroj et al. [51] found that Clinpro could inhibit *S*. *mutans* growth when applied to paper and enamel disks using agar diffusion assays, but showed a reduced effect in a planktonic growth inhibition assay. Contrarily, Matalon et al. [52] showed that none of the four fluoride-releasing pit-and-fissure sealants tested showed any antibacterial activity after a 30-day aging process. Similarly, previous studies showed that another fluoride-releasing resin-based sealant, Helioseal F, failed to show detectable bactericidal activity both in a film contact test [53] and a disk diffusion test [54], which is in accordance with our present results. These findings are also consistent with the results of a previous investigation, in which Clinpro aged for 7 days failed to cause zones of inhibition against *S*. *mutans* using an agar diffusion method [20]. We speculated that differences in test methods and sample preparation procedure might be the main factor contributing to the contradictory results. The sustained release of fluoride ions from fluoride-containing materials could be a substantial benefit for caries inhibition, because fluoride could reduce demineralization, enhance remineralization of the enamel and dentin, and inhibit the metabolism of bacteria at concentrations of 10 ppm or higher[27]. However, sustaining release of fluoride in different forms or vehicles could not reach the expected level. Therefore, the inhibitory effect generated by fluoride on acid production of biofilms was negligible; fluoride alone could not prevent the formation of the biofilm on dental surfaces [27].

In recent years, the antibacterial modification of resin materials with QAMs has become a key topic of research in the field of dental materials. The antibacterial activity of QAMs can be attributed to their positively charged quaternary amine N+, which can make contact with the negatively charged bacterial cell, disturb the electric balance, disrupt the cell membrane, and cause cytoplasmic leakage [50, 55, 56]. Although QAMs such as MDPB and DMAE-CB monomers could chemically bond to the resin matrix and exert stable antibacterial activity, Li et al.[57] and Ebi et al.[58] reported that the amount of MDPB incorporated should be limited in order to maintain the stability of the parent material. There is only one double bond in the chemical structures of MDPB and DMAE-CB, which renders the resins with limited antibacterial activity. By contrast, the MAE-DB monomer contains two carbon-carbon double bonds, and thus a higher amount of the polymerizable antibacterial monomer could be incorporated into resin materials with less of a negative impact on the physical and mechanical properties [59]. According to the CLSM results, the number of adherent cells was lowest on the MAE-DB-incorporated sealants, and the biofilms consisted of primarily dead bacteria. Our previous study showed that an uncured MAE-DB monomer exhibited strong bactericidal action against oral bacteria [59], and MAE-DB-incorporated resin could inhibit bacterial adhesion and caries generation by attenuating *gftB* expression and impairing the membrane integrity of *S*. *mutans* [43].

In summary, the results of the present study show that incorporation of MAE-DB into a resin sealant could render the parent material with short-term and long-term anti-*S*. *mutans* activity, indicating MEA-DB as a good candidate for endowing resin-based pit-and-fissure sealants with antibacterial properties. Assessments of the physical and chemical properties of MAE-DB-containing sealants such as surface contact angle, degree of conversion, microhardness, compressive strength, microleakage, and chemical stabilities are currently in progress.

## Supporting Information

S1 File Raw Data. (XLSX)
